# Association of Triglyceride Deposit Cardiomyovasculopathy With Drug-Eluting Stent Restenosis Among Patients With Diabetes

**DOI:** 10.1001/jamanetworkopen.2020.12583

**Published:** 2020-08-07

**Authors:** Yusuke Nakano, Mayu Suzuki, Ken-ichi Hirano, Hirohiko Ando, Hiroaki Takashima, Hiroshi Takahashi, Tetsuya Amano

**Affiliations:** 1 Department of Cardiology, Aichi Medical University, Nagakute, Aichi, Japan; 2 Laboratory of Cardiovascular Disease, Novel, Non-invasive, and Nutritional Therapeutics, Triglyceride Research Center, Osaka University Graduate School of Medicine, Suita, Osaka, Japan; 3 Fujita Health University School of Medical Science, Kutsukake, Toyoake, Aichi, Japan

## Abstract

This cohort study uses data from a single-center cardiac catheter database to investigate the association of cardiac stent restenosis in patients with diabetes mellitus and comorbid triglyceride deposit cardiomyovasculopathy.

## Introduction

Type 2 diabetes remains an independent risk factor for vascular failure even in the setting of advanced medical treatment approaches and technologies for vascular disease, such as percutaneous coronary intervention. Triglyceride deposit cardiomyovasculopathy (TGCV) is a rare cardiovascular disorder caused by defective hydrolysis of intracellular triglycerides and newly encoded as an orphan disease in Europe in 2019 (Orphanet ORPHAcode No. 565612).^1,2^ Orphan diseases are rare diseases that affect only a small number of individuals. TGCV is characterized by diffuse narrowing of the coronary arteries due to atherosclerosis with triglyceride deposits within the endothelial and smooth muscle cells.^1,2^ It is well known that diabetes leads to cellular TG deposition^3^, and our previous postmortem study revealed that some patients with diabetes who had died of cardiovascular diseases exhibited the TGCV phenotype.^4^ Therefore, we investigated the extent to which a TGCV comorbidity is associated with outcomes after percutaneous coronary intervention in patients with diabetes.

## Methods

This retrospective single-center cohort study included data from the cardiac catheter database at Aichi Medical University Hospital between May 1, 2010, and March 31, 2018. Among 526 consecutive patients with diabetes implanted with second-generation drug-eluting stents (DESs), which are a type of medical device used in percutaneous coronary intervention, data from 81 patients were allocated to either the TGCV group or the control group for subsequent comparative analyses (eFigure and eTable in the Supplement). The primary end point was a binary in-stent restenosis (ISR) assessed using quantitative coronary angiography. The secondary end points were an in-stent late loss, a parameter used to quantify the degree of neointimal hyperplasia after coronary stenting, a target lesion revascularization, and an evaluation of angiographic ISR morphology. Follow-up coronary angiography was usually performed 8 to 12 months after percutaneous coronary intervention (eMethods in the Supplement). The study protocol was approved by the ethics committee of Aichi Medical University, and written informed consent was obtained from all patients and their families. This report follows the Strengthening the Reporting of Observational Studies in Epidemiology (STROBE) reporting guideline.

Continuous variables were analyzed using *t* tests and the Mann-Whitney *U* test; categorical data were analyzed using Fisher exact test. In-stent late loss between the 2 groups was compared using analysis of covariance. Univariate and multivariate logistic regression analyses were performed to study the influence of TGCV on outcomes. Statistical significance was set at a 2-tailed *P* value <.05. SPSS Statistics software, version 25.0 (IBM Corp), was used for statistical analyses.

## Results

This cohort study included a total of 81 patients: 7 and 74 patients with and without TGCV, each of them having 15 and 111 lesions, respectively. Overall, the mean (SD) age was 66 (11) years; 63 were men (77.8%) and 18 were women (22.2%) Demographics include Asian race/ethnicity: 7 (100%) in the TGCV group vs 74 (100%) in the control group (*P* > .99). (Table). In-stent late loss was greater in the TGCV group than in the control group (0.91 mm [interquartile range, 0.27-2.39 mm] vs 0.15 mm [interquartile range, 0.03-0.35 mm]; *P* < .001) (Figure), with a higher incidence of binary ISR and target lesion revascularization in the former than in the latter group (46.7% vs 9.0% [*P* < .001] and 33.3% vs 6.3% [*P* = .006], respectively). Multivariate analysis showed that TGCV was significantly and independently associated with ISR after DES implantation (odds ratio, 5.31; 95% CI, 1.32-21.4; *P* = .02). Diffuse or occlusive restenosis patterns were more frequent in the TGCV group than in the control group (100.0% vs 20.0%; *P* = .003).

**Table.** Patients Characteristics, Lesion Characteristics, and QCA and Restenosis Data^a^

Variables	TGCV	Control	*P* value
Patients characteristics			
No. of patients	7	74	
Men	5 (71)	58 (78)	.65
Age, mean (SD), y	66.3 (12.8)	65.6 (10.6)	.88
Race/ethnicity, Asian	7 (100)	74 (100)	>.99
BMI, mean (SD), kg/m^2^	22.9 (3.9)	24.2 (4.0)	.41
BMIPP wash-out, mean (SD), %	2.8 (6.0)	25.9 (10.5)	<.001
Hypertension	6 (86)	40 (54)	.13
Dyslipidemia	6 (86)	49 (66)	.42
Current smoker	2 (29)	25 (34)	>.99
Prior MI	3 (43)	9 (12)	.06
Hemodialysis	2 (29)	5 (7)	.11
Duration of diabetes, median (IQR), y	7 (1-11)	9 (1-13)	.84
HbA_1c_, mean (SD), %			
Baseline	7.3 (1.8)	7.2 (1.4)	.92
1-y follow-up	6.9 (0.8)	6.9 (1.1)	.98
Insulin	3 (43)	9 (12)	.06
Statin use	6 (86)	71 (96)	.31
DAPT	7 (100)	73 (99)	>.99
P2Y12 inhibitor use	7 (100)	71 (96)	>.99
Lesion characteristics			
No. of lesions	15	111	
Target vessel			
RCA	6 (40)	30 (27)	.59
LAD	6 (40)	56 (51)	
LCX	3 (20)	25 (23)	
ACC/AHA type B2/C	9 (60)	96 (87)	.02
Bifurcation lesion	8 (53)	47 (42)	.58
DES type^b^			
XIENCE	8 (53)	76 (69)	.008
SYNERGY	0 (0)	14 (13)	
Resolute	1 (7)	9 (8)	
Endeavor	5 (33)	4 (4)	
Nobori	1 (7)	4 (4)	
Ultimaster	0 (0)	4 (4)	
Stent			
Diameter, mean (SD), mm	2.88 (0.36)	3.05 (0.42)	.14
Length, median (IQR), mm	18.0 (15.0-23.0)	23.0 (16.0-30.0)	.07
QCA data, mean (SD)			
Lesion length, mm	20.1 (12.8)	20.0 (9.6)	.99
Reference diameter, mm	2.63 (0.62)	2.76 (0.60)	.44
Pre			
MLD, mm	0.76 (0.48)	0.92 (0.46)	.24
%DS, %	69.8 (17.4)	72.9 (19.0)	.58
Post			
MLD, mm	2.61 (0.54)	2.61 (0.46)	.99
%DS, %	3.9 (7.7)	8.2 (10.4)	.12
Follow-up			
MLD, mm	1.41 (0.87)	2.28 (0.68)	<.001
%DS, median (IQR), %	31.0 (10.0-82.0)	13.0 (8.0-21.0)	.02
Duration to follow-up CAG, median (IQR), mo	10.1 (6.6-13.2)	9.1 (7.0-11.0)	.39
Vascular failure			
ISR	7 (47)	10 (9)	<.001
TLR	5 (33)	7 (6)	.006
Restenosis morphology			
Diffuse or occlusive type	7 (100)	2 (20)	.003

Abbreviations: ACC/AHA, American College of Cardiology/American Heart Association; BMI, body mass index; BMIPP, ^123^I-β-methyl iodophenyl-pentadecanoic acid; CAG, coronary angiography; DAPT, dual antiplatelet therapy; DES, drug-eluting stent; DS, diameter stenosis; HbA_1c_, hemoglobin A_1c_; IQR, interquartile range; ISR, in-stent restenosis; LAD, left anterior descending artery; LCX, left circumflex artery; MI, myocardial infarction; MLD, minimal lumen diameter; QCA, quantitative coronary angiography; RCA, right coronary artery; TGCV, triglyceride deposit cardiomyovasculopathy; TLR, target lesion revascularization.

^a^ Values are written as No. (%) unless otherwise specified.

^b^ Manufacturers of the DESs are as follows: XIENCE (Abbott Vascular), SYNERGY (Boston Scientific), Resolute (Medtronic), Endeavor (Medtronic), Nobori (Terumo Corporation), Ultimaster (Terumo Corporation).

**Figure.**
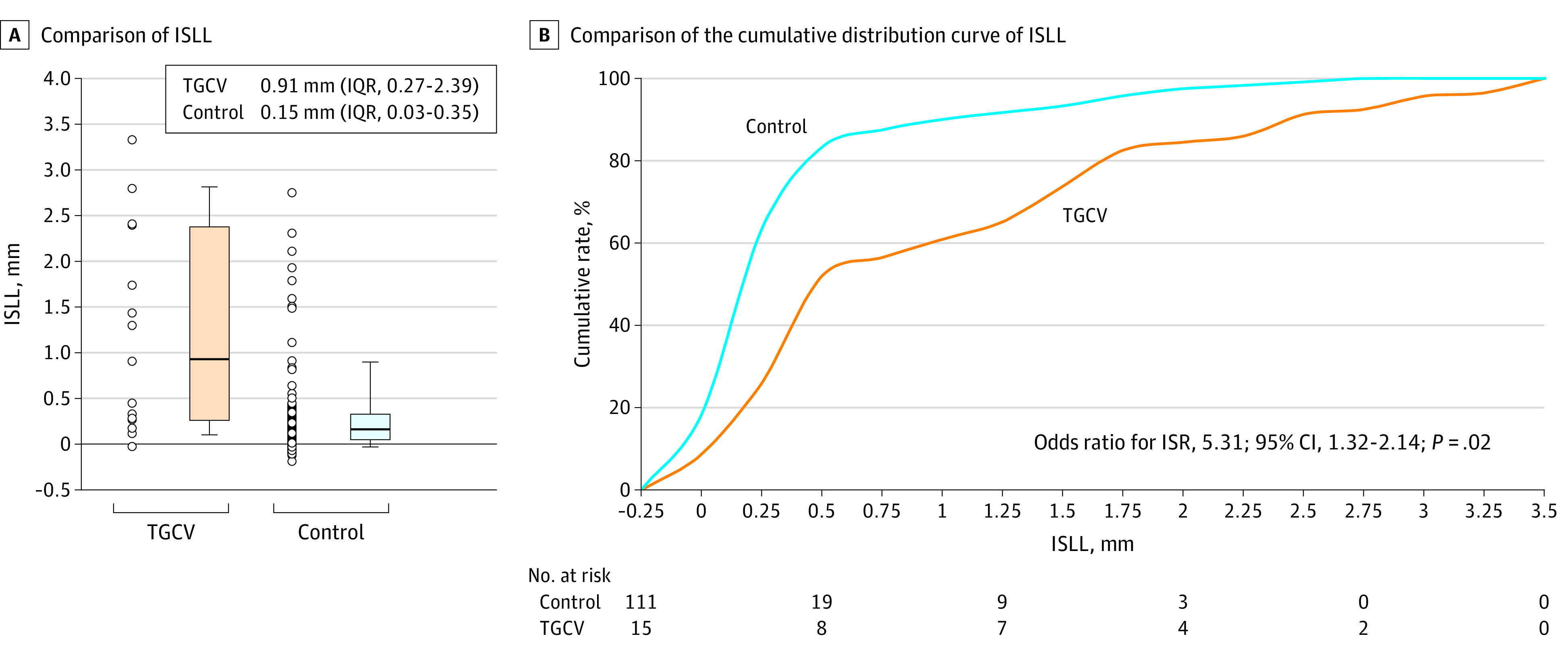
Comparison of ISLL and the Cumulative Distribution Curve of ISLL Between the TGCV and Control Groups A. ISLL, a parameter determined by caliper measurements using the known diameter of the angiographic catheter as a reference, was significantly higher in the TGCV group than in the control group. B. The cumulative distribution of ISLL revealed a greater value of ISLL in the TGCV group than in the control group. *P* value was calculated using ANCOVA with the following variables: American College of Cardiology/American Heart Association classification type B2/C, DES type, and TGCV diagnosis. ANCOVA indicates analysis of covariance; DES, drug-eluting stent; ISLL, in-stent late loss; IQR, interquartile range; ISR, in-stent restenosis; TGCV, triglyceride deposit cardiomyovasculopathy. *P* < .0001 vs control.

## Discussion

To our knowledge, this is the first study to assess the association of TGCV with 1-year outcomes after second-generation DES implantation. Study results suggest that a higher in-stent late loss at the 1-year follow-up, with a subsequently increased rate of ISR, was observed in the TGCV group than in the control group. Furthermore, TGCV was found to be independently associated with ISR even after adjustment for confounding factors. Diffuse or occlusive restenosis morphology was more frequently observed in the TGCV group than in the control group. The plausible mechanisms for the clinical deficiency of DESs in the setting of TGCV may be associated with an increased amount of inflammatory cytokines and growth factors that are expressed in TGCV mouse models.^5^ Furthermore, mammalian target of rapamycin inhibitors used in DESs might locally affect lipolysis in triglyceride-harboring smooth muscle cells and other vascular cells within the target lesion in TGCV.^6^ This study has several limitations, including potential bias due to the retrospective nature of the analysis and a small number of patients with TGCV resulting in the multivariate analysis being potentially overadjusted.

In conclusion, comorbidity of TGCV with diabetes was associated with an increased incidence of vascular failure. These findings warrant future prospective clinical studies with larger cohorts.

## Notes

Supplement. **eMethods.** Supplemental Methodological Descriptions **eFigure.** Patient Flow and Informed Consent **eTable.** The Diagnostic Criteria for TGCV **eReferences**

## References

[1] Hirano K, Ikeda Y, Zaima N, Sakata Y, Matsumiya G Triglyceride deposit cardiomyovasculopathy. N Engl J Med. 2008;359(22):2396-2398. doi:10.1056/NEJMc0805305 19038890

[2] Li M, Hirano KI, Ikeda Y, ; Japan TGCV study group Triglyceride deposit cardiomyovasculopathy: a rare cardiovascular disorder. Orphanet J Rare Dis. 2019;14(1):134. doi:10.1186/s13023-019-1087-4 31186072 PMC6560904

[3] Greenberg AS, Coleman RA, Kraemer FB, The role of lipid droplets in metabolic disease in rodents and humans. J Clin Invest. 2011;121(6):2102-2110. doi:10.1172/JCI46069 21633178 PMC3104768

[4] Ikeda Y, Zaima N, Hirano K, Coronary triglyceride deposition in contemporary advanced diabetics. Pathol Int. 2014;64(7):325-335. doi:10.1111/pin.12177 25047503

[5] Lin Y, Chiba S, Suzuki A, Vascular smooth muscle cells isolated from adipose triglyceride lipase-deficient mice exhibit distinct phenotype and phenotypic plasticity. Biochem Biophys Res Commun. 2013;434(3):534-540. doi:10.1016/j.bbrc.2013.03.109 23583398

[6] Singh R, Kaushik S, Wang Y, Autophagy regulates lipid metabolism. Nature. 2009;458(7242):1131-1135. doi:10.1038/nature07976 19339967 PMC2676208

