# Supplementary material for: Association of Triglyceride Deposit Cardiomyovasculopathy With Drug-Eluting Stent Restenosis Among Patients With Diabetes
Source: JAMA Netw Open. 2020 Aug 7;3(8):e2012583. doi: 10.1001/jamanetworkopen.2020.12583 (PMC7414388; doi:10.1001/jamanetworkopen.2020.12583)
Supplement: Supplement. — eMethods. Supplemental Methodological Descriptions eFigure. Patient Flow and Informed Consent eTable. The Diagnostic Criteria for TGCV eReferences [file jamanetwopen-3-e2012583-s001.pdf]

Supplementary Online Content

Nakano Y, Suzuki M, Hirano K, et al. Association of triglyceride deposit cardiomyovasculopathy with drug-eluting stent restenosis among patients with diabetes. *JAMA Netw Open*. 2020;3(8):e2012583. doi:10.1001/jamanetworkopen.2020.12583

eMethods. Supplemental Methodological Descriptions

eFigure. Patient Flow and Informed Consent

eTable. The Diagnostic Criteria for TGCV

eReferences

This supplementary material has been provided by the authors to give readers additional information about their work.

© 2020 Nakano Y et al. *JAMA Network Open.*

eMethods. Supplemental Methodological Descriptions

Description of patients flow and Informed consent (eFigure.)

The latest diagnostic criteria for TGCV was published in 2018 by the Japan TGCV study group which is a governmental rare disease project in Japan. Based on this, a total of 526 consecutive patients with DM implanted 2nd generation DESs between 2010 and 2018 were retrospectively assessed for eligibility. Informed consent was obtained from all of the 526 patients. Of which, 424 patients were excluded mainly due to the insufficient data required for the diagnosis of TGCV including those from BMIPP myocardial scintigraphy. Finally, data from 81 were extracted, and retrospectively allocated to 7 patients diagnosed with definitive TGCV and 74 non-TGCV controls.

Description of the diagnostic criteria for TGCV (eTable.)

The latest version of the diagnostic criteria for TGCV comprises four items, in which 4 points or more indicate a definitive diagnosis of TGCV. The two major items refer to triglyceride deposition in the myocardium and coronary arteries. The two minor items are Jordans' anomaly and diabetes mellitus (DM).[1,2] DM diagnosis is based on the diagnostic criteria of DM by the Japan Diabetes Society.[3]

Description of assessment of clinical outcomes

Endpoints

- The primary endpoint: binary in-stent restenosis (ISR).

ISR was defined as angiographic luminal diameter narrowing >50% anywhere within the stent and/or within the 5-mm borders proximal or distal to the stent, as assessed using quantitative coronary angiography (QCA). Follow-up coronary angiography was usually performed 8–12 months after PCI in patients without the evidence of restenosis.

- The secondary endpoints: in-stent late loss (ISLL), target lesion revascularisation (TLR), and evaluation of angiographic ISR morphology.

ISLL, a parameter to quantify the degree of neointimal hyperplasia after coronary stenting, was defined as the difference between the minimal lumen diameter (MLD) immediately after PCI and the MLD at follow-up angiography.
TLR was defined as revascularisation of the target lesion after either PCI or coronary artery bypass grafting on the basis of clinical or physiological assessment. Clinically driven TLR was considered if re-intervention of the target lesion was required due to the presence of a symptomatic stenosis of ≥50% of the diameter during follow-up. Ischaemia-driven TLR was considered if re-intervention was performed on a patient with ischaemic signs examined using noninvasive tests or fractional flow reserve assessment, with an inducible ischaemia cut-off of 0.75.
Angiographic ISR morphology was evaluated according to the Mehran classification: focal ISR pattern or diffuse or occlusive ISR pattern.[4] Coronary lesion types were based on the American Heart Association/American College of Cardiology (AHA/ACC) classification.

QCA analysis
Contrast-filled guide catheters were used as the reference standard in QCA. Matched end-diastolic frames of the angiograms before and after PCI and at follow-up were analysed by contour detection using the minimum cost algorithm QCA-CMS (version 3.0, MEDIS, Leiden, The Netherlands). Cine frames for catheter calibration or vessel QCA analysis were selected by an experienced QCA analyst (K.Y.). All vessel measurements for the selected frames were performed by two experienced QCA analysts with similar laboratory experience (K.Y. and Y.N.).

Statistical analysis
Continuous variables with normal distribution were expressed as means ± standard deviation, and between-group comparisons were performed using unpaired Student's *t* test. Normality of the variables was assessed, and those failing the normality tests were expressed as medians with interquartile ranges (IQRs) and compared using Mann–Whitney *U* test. Categorical variables were presented as patient number (%) and analysed using Fisher's exact test. ISLL was compared between the two groups using analysis of covariance. Multivariate logistic regression analysis was performed to assess the predictors of ISR and TLR adjusted for sex, age, and all variables with a p value < 0.10 in univariate analysis. A p value < 0.05 was considered to indicate significance. All statistical analyses were performed using SPSS Statistics software, version 25.0 (IBM Corp).

© 2020 Nakano Y et al. *JAMA Network Open.*

eFigure. Patient flow and Informed consent

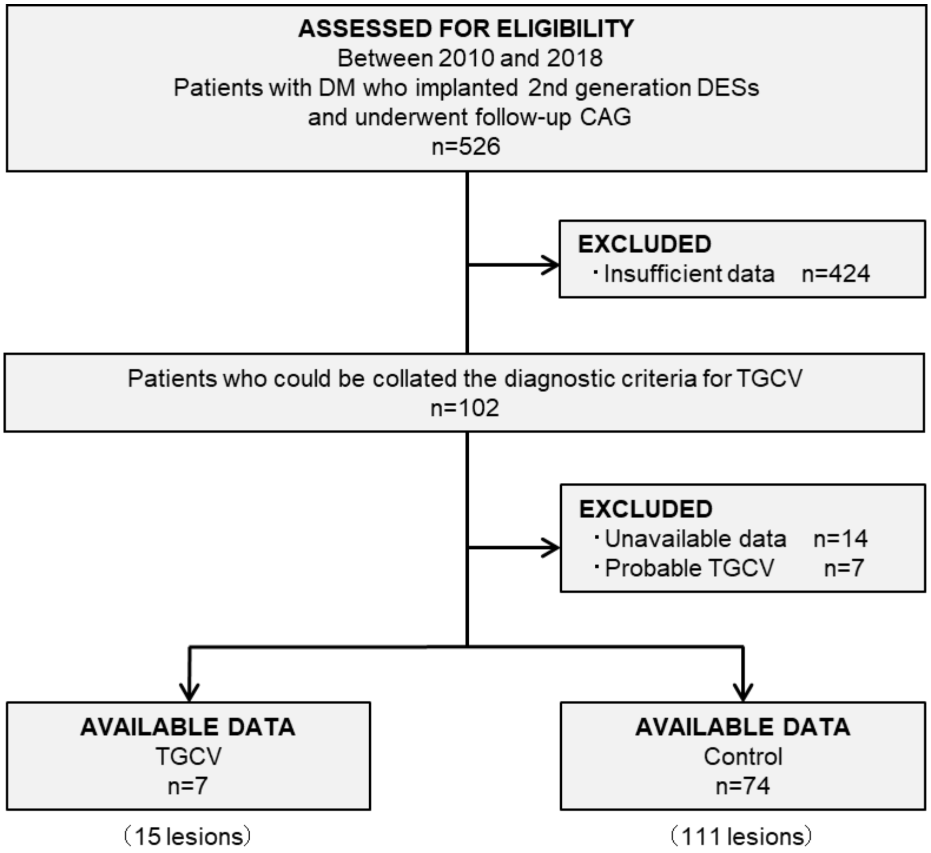

DM, diabetes mellitus; DESs, drug-eluting stents; CAG, coronary angiography; TGCV, triglyceride deposit cardiomyovasculopathy

© 2020 Nakano Y et al. *JAMA Network Open.*

eTable. The diagnostic criteria for TGCV

Items	Clinical findings	
1. Major items (2 points)	1.1 Myocardial TG deposition or impaired LCFA metabolism	
	At least one of the following:	
	1) Myocardial TG deposition by biopsy specimens	
	2) Myocardial TG deposition by MR spectroscopy	
	3) Decreased washout rate (<10%) of BMIPP	
	1.2 Diffuse narrowing coronary arteries demonstrated by CAG, CT angiography	
2. Minor items (1 point)	2.1 Jordans‘ anomaly (apparent vacuoles of about 1 micrometer in size) of polymorphonuclear leucocytes in peripheral blood smear	
	2.2 Diabetes mellitus	
Diagnosis of TGCV	(1) 4 points or more	Definite TGCV
	(2) 3 points	Probable TGCV

TGCV, triglyceride deposit cardiomyovasculopathy; TG, triglyceride; LCFA, long-chain fatty acid; MR, magnetic resonance; BMIPP, $[^{123}I]$-β-methyl iodophenyl-pentadecanoic acid; CAG, coronary angiography; CT, computed tomography.

© 2020 Nakano Y et al. *JAMA Network Open.*

eReferences.

1. Miyauchi H, Hashimoto C, Ikeda Y, et al. Diagnostic Criteria and Severity Score for Triglyceride Deposit Cardiomyovasculopathy. *Annals of Nuclear Cardiology.* 2018;4(1):94-100.
2. Jordans GH. The familial occurrence of fat containing vacuoles in the leukocytes diagnosed in two brothers suffering from dystrophia musculorum progressiva (ERB.). *Acta Med Scand.* 1953;145(6):419-423.
3. Seino Y, Nanjo K, Tajima N, et al. Report of the Committee on the classification and 44 diagnostic criteria of diabetes mellitus. *Diabetology International*. 2010;1(1):2 20.
4. Mehran R, Dangas G, Abizaid AS, et al. Angiographic patterns of in-stent restenosis: classification and implications for long-term outcome. *Circulation.* 1999;100(18):1872-1878.

© 2020 Nakano Y et al. *JAMA Network Open.*
